# Alterations of Gut Microbiota by Overnutrition Impact Gluconeogenic Gene Expression and Insulin Signaling

**DOI:** 10.3390/ijms22042121

**Published:** 2021-02-20

**Authors:** Ling He

**Affiliations:** Departments of Pediatrics and Pharmacology & Molecular Sciences, Johns Hopkins University School of Medicine, Baltimore, MD 21287, USA; heling@jhmi.edu

**Keywords:** overnutrition, microbiota, lipopolysaccharide, acetyltransferase P300, gluconeogenic gene, insulin resistance

## Abstract

A high-fat, Western-style diet is an important predisposing factor for the onset of type 2 diabetes and obesity. It causes changes in gut microbial profile, reduction of microbial diversity, and the impairment of the intestinal barrier, leading to increased serum lipopolysaccharide (endotoxin) levels. Elevated lipopolysaccharide (LPS) induces acetyltransferase P300 both in the nucleus and cytoplasm of liver hepatocytes through the activation of the IRE1-XBP1 pathway in the endoplasmic reticulum stress. In the nucleus, induced P300 acetylates CRTC2 to increase CRTC2 abundance and drives *Foxo1* gene expression, resulting in increased expression of the rate-limiting gluconeogenic gene *G6pc* and *Pck1* and abnormal liver glucose production. Furthermore, abnormal cytoplasm-appearing P300 acetylates IRS1 and IRS2 to disrupt insulin signaling, leading to the prevention of nuclear exclusion and degradation of FOXO1 proteins to further exacerbate the expression of *G6pc* and *Pck1* genes and liver glucose production. Inhibition of P300 acetyltransferase activity by chemical inhibitors improved insulin signaling and alleviated hyperglycemia in obese mice. Thus, P300 acetyltransferase activity appears to be a therapeutic target for the treatment of type 2 diabetes and obesity.

## 1. Dietary Composition Shapes Gut Microbiota

Immediately after birth, the baby acquires bacteria from the mother and surrounding environment. The intestinal tract is quickly colonized by bacteria, and the bacteria metabolize nutrients, producing high levels of lactate and acetate in the intestine [1,2]. Subsequently, gut microbiota begin to affect the host’s life and proceed in an incremental manner from infancy to adulthood, thus playing a critical role in maintaining intestinal functions and the host’s health [3,4]. It is estimated that there are 100 trillion bacteria live in the gastrointestinal tract, and the total bacterial genes are 150-fold that of the human genome [5]. In the intestine, there are four major microbial phyla: *Firmicutes*, *Bacteroides*, *Proteobacteria,* and *Actinobacteria*, that account for over 90% of the gut microbiota, of which *Bacteroides* are considered good bacteria [6,7]. The composition of gut microbiota is highly dynamic during the development, and its dysbiosis is implicated in different health and disease conditions. Nutrients from food are the principal energy source to maintain the growth of gut microbiota. Changes in dietary patterns can lead to up to 57% of gut microbiota changes and reshape the population of gut microbiota [8,9]. It is considered that dietary composition is the most influential factor in shaping and affecting gut microbiota composition [10].

## 2. Overnutrition Leads to Changes in the Composition of Gut Microbiota

Obesity has become a global pandemic due to excessive consumption of nutrients and a sedentary lifestyle. Patients with type 2 diabetes (T2D) and obesity have a different composition of gut microbiota, and gut microbial dysbiosis is associated with obesity and T2D [11,12]. A high-fat, Western-style diet is an important predisposing factor for the onset of diabetes and obesity, and it is clear that the consumption of a Western diet with high calorie content has contributed to the obesity pandemic [13,14]. High-fat-diet (HFD) feeding can change the gut microbial profile and reduce its diversity. Specifically, HFD feeding leads to a reduction of *Bacteroides* and an increase in *Firmicutes* [15,16,17]. Cumulative evidence shows that *Firmicutes* dominate the gut microbiota in obese patients [18]. Furthermore, a sugar-enriched diet also causes a significant shift in the gut microbiota in animals [19]. In a study, over 200 strains of mice were used to determine the effects of dietary factors on gut microbiota and reported that feeding a high-fat and high-sugar diet reproducibly altered gut microbiota despite differences in the host genotype [20]. There is a linear dose response between the gut microbiota and dietary perturbations, and a new steady state of the bacterial group is reached after 3.5 days of each dietary perturbation. Germ-free mice that received a fecal transfer from either obese mice or obese patients led to an increase in body fat [11,21]. Germ-free mice with a transfer of the gut microbiota from obese-prone mice, not the obese-resistant mice, developed obesity, increased gut permeability, and inflammation [22]. However, of importance, most diet-induced changes in gut microbiota are reversible [20].

## 3. Overnutrition Elevates Serum Lipopolysaccharide Levels

HFD-induced obesity and metabolic disorders are associated with increased blood bacterial lipopolysaccharide (LPS) levels and its initiated low grade of inflammation [23]. It has been documented that mice fed an HFD for as short a period of time as 2 weeks exhibited a significant increase in serum LPS levels [23]. However, LPS from different microbial phyla has a distinct structure and exerts either a potent activator or an innate immune inhibitor [24,25]. LPS from genus *Bacteroides* harbor tetra- and penta-acylated lipid A structures compared to the hexa-acylated lipid A structure found in other microbial phyla, making the LPS from genus *Bacteroides* an antagonist of immune stimulation and inflammatory cytokine response [24,25]. This beneficial effect is lost when fed an HFD (range from 44–72% calories) as a result of HFD-induced reduction of *Bacteroides* along with increased other microbial phyla, such as *Firmicutes* [15,16,18,26]. This should result in an exaggerated LPS-initiated immune response.

**Figure 1:**
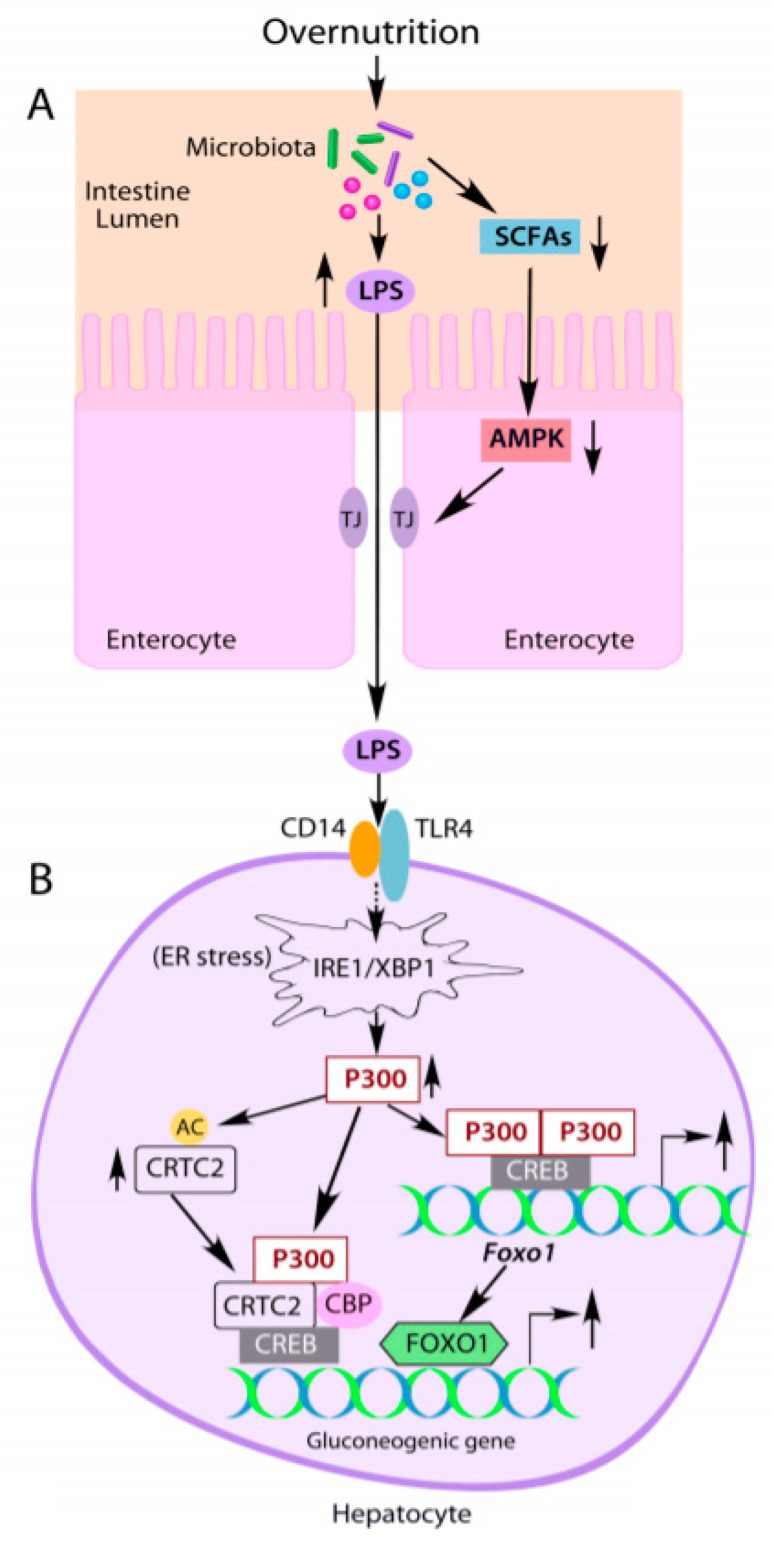
Overnutrition leads to increased gluconeogenic gene expression by altering gut microbiota and lipopolysaccharide (LPS) production. (**A**) Overnutrition alters the composition of gut microbiota and increases LPS production along with decreased short chain fatty acid (SCFA) levels. Decreased SCFAs lead to the impairment of AMPK activity in intestinal epithelial cells, resulting in leaky gut and endotoxemia. (**B)** Elevated LPS induces acetyltransferase P300 via activation of the IRE1-XBP1 pathway in the endoplasmic reticulum (ER) stress in hepatocytes. Induced P300 upregulates gluconeogenic gene expression by directly binding to CREB, acetylating CRTC2 to prevent its nuclear exclusion and degradation, and driving *Foxo1* gene expression. FOXO1 can bind to the promoter of the gluconeogenic gene to further increase gluconeogenic gene expression. TJ: tight junction. The solid arrows indicate the direct effects, and the dashed arrow indicates indirect effects.

In the intestine, microbiota can metabolize glucose, amino acids, and organics to generate short chain fatty acids (SCFAs), such as lactate, butyrate, and propionate. SCFAs can regulate the homeostasis of human health and the progression of diseases [27,28] and have been used to prevent and treat metabolic diseases [29,30]. SCFAs are important fuels for intestinal epithelial cells and can strengthen the intestinal barrier and maintain intestinal integrity by activating AMP-activated kinase (AMPK). Activated AMPK can affect the formation of tight junctions to maintain intestinal barrier integrity, resulting in decreased LPS leakage from the gut [31,32]. However, the reduction of SCFAs by HFD feeding [33] can lead to the impairment of the intestinal barrier and an increase in intestinal permeability (leaky gut] in obese mice and diabetic patients by impairing AMPK (Figure 1A) [34,35]. Collectively, HFD feeding causes shifts in the composition of the gut microbiota and increases the intestinal permeability and LPS leakage, and its initiated low-grade inflammation.

## 4. LPS-induced Acetyltransferase P300 Upregulates Liver Gluconeogenesis in Obesity

### 4.1. P300 Plays an Important Role in Regulating Glucose Metabolism

Glucose is a major source of energy for mammalian cells and takes a central position in metabolism. Several mammalian tissues and cells, such as neurons, erythrocytes, and renal medulla, use glucose as the sole energy source. Low blood glucose levels (hypoglycemia) cause damage to these tissues and cells, even death [36,37]. Therefore, a continuous supply of glucose as a source of metabolic energy is required for maintaining normal functions and imposes that blood glucose levels should not fall below ~5 mM euglycemic levels in healthy individuals. In response to the decrease in blood glucose levels, counterregulatory mechanisms are activated by rapidly inducing the secretion of glucagon, epinephrine, and glucocorticoids to increase liver glucose production directly or indirectly through the cAMP-PKA signaling pathway [38]. Activated PKA phosphorylates cAMP-response element-binding protein (CREB) at S133, recruiting co-activators CBP, P300, and CRTC2 to form the gluconeogenic engine to CRE containing rate-limiting gluconeogenic gene *Pck1* and *G6pc,* and driving liver gluconeogenesis [39,40,41,42,43].

CBP and its paralogue P300 share extensive homology. Both have three cysteine-histidine-rich domains (CH1–3), the bromodomain, the CREB binding Kix domain, the histone acetyltransferase domain, and the SID domain [44,45]. The acetylation of histones by CBP and P300′s acetyltransferase activity results in chromatin remodeling and transcriptional activation, and their associations with basal transcription factors and RNA polymerase II as well as a variety of other transcription factors bridges the basal transcription machinery and upstream transcription factors, facilitating the transcription of the target gene [44,45]. P300 can acetylate CRTC2 at K628 to prevent CRTC2 degradation [46]. Both P300 and CBP can acetylate other proteins such as FXR, SREBP-1, and FOXO1 to modulate gene expression related to glucose and lipid metabolism [47,48,49].

The gluconeogenic pathway accounted for 50–70% of newly synthesized glycogen [50], and physiologic concentration of glucose cannot effectively promote the glycogen synthesis when glucose was the sole substrate; however, efficient glycogen synthesis occurred when gluconeogenic precursors were added [51]. It has been proven that the gluconeogenic pathway contributes substantially to hepatic glycogen formation during the post-prandial state [52,53]. We found that depletion of liver P300 decreased glycogen storage in the liver, leading to relative hypoglycemia [54]. The phosphorylation of CBP at S436 leads to disassembly of CBP and CRTC2, not P300, from CREB [55]. Interestingly, P300 lacks the corresponding S436 phosphorylation site found in CBP. We generated a phosphorylation-competent P300G422S knock-in mouse model and found that mutant mice exhibited reduced liver glycogen content and produced significantly less glycogen in a tracer incorporation assay in the postprandial state, demonstrating an important and unique role of P300 in glycogen synthesis through maintaining basal gluconeogenesis [54].

### 4.2. Induction of Liver P300 by LPS Leads to Abnormal Glucose Production in Obesity

Patients with T2D and obesity exhibit abnormal liver glucose production, the major cause of fasting hyperglycemia [56,57,58]. Considering the importance of P300 and CBP in the regulation of glucose metabolism, we determined the protein levels of P300 and CBP in the liver of mice fed an HFD for different periods of time and found that the protein levels of P300, not CBP, were dramatically induced by an HFD after only one week of feeding, an event that occurred prior to the occurrence of insulin resistance [59]. Obese *ob/ob* mice also have significantly elevated P300 protein levels in the liver. Since increased LPS levels can initiate a low-grade of inflammation and endoplasmic reticulum (ER) stress in T2D and obesity, we examined LPS levels in the liver and found that LPS levels were significantly increased in the liver of HFD-fed mice. Most importantly, the LPS treatment increased P300 protein levels both in the nucleus and cytoplasm in cultured hepatocytes and in the liver of mice. HFD feeding could not induce P300 in the liver of CD14 knockout mice, suggesting that the induction of liver P300 is through the TLR/CD14 pathway.

ER stress is the responsible site for protein synthesis, maturation, and transport. Many disturbances cause accumulation of unfolded proteins in the ER and trigger an evolutionarily conserved response, termed the unfolded protein response (UPR), leading to the activation of three canonical pathways: inositol-requiring enzyme 1(IRE1)-XBP1s, PKR-like ER-regulating kinase (PERK), and activating transcription factor 6 (ATF6) [60]. We and others found that LPS treatment can activate ER stress [59,61], and the depletion of IRE1 and XBP1 blocked P300 induction by LPS, indicating P300 induction through the activation of the IRE1-XBP1s pathway (Figure 1B). Further studies revealed that LPS treatment significantly decreased ubiquitin conjugated P300 in Hepa1-6 cells; thus, LPS induces P300 by decreasing its ubiquitination and degradation [59].

Moreover, in HFD-fed mice, depletion of liver P300 by shRNA significantly decreased liver glucose production, and the inhibition of P300 acetyltransferase by C646 or A-485 [62,63,64,65] significantly reduced glucose production in primary hepatocytes and the mRNA levels of *G6pc* [59,66]. Since the acetylation of CRTC2 by nuclear P300 reduces CRTC2 degradation [46], elevated P300 protein levels could augment gluconeogenic gene expression by increasing CRTC2 protein levels in HFD-fed mice (Figure 1B). Additionally, FOXO1 upregulates gluconeogenesis through activation of *Pck1* and *G6pc* gene expression in the liver [67,68]. P300 can regulate the expression of the *Foxo1* gene through binding to tandem cAMP-response element sites in the proximal promoter region of the *Foxo1* gene [62]. Elevated nuclear P300 should also increase the expression of the *Foxo1* gene in the liver of obese mice (Figure 1B). These data indicate that P300 is an important etiological factor for abnormal glucose production in the liver of T2D and obesity.

## 5. LPS-Initiated Low-Grade Inflammation Causes Insulin Resistance

**Figure 2:**
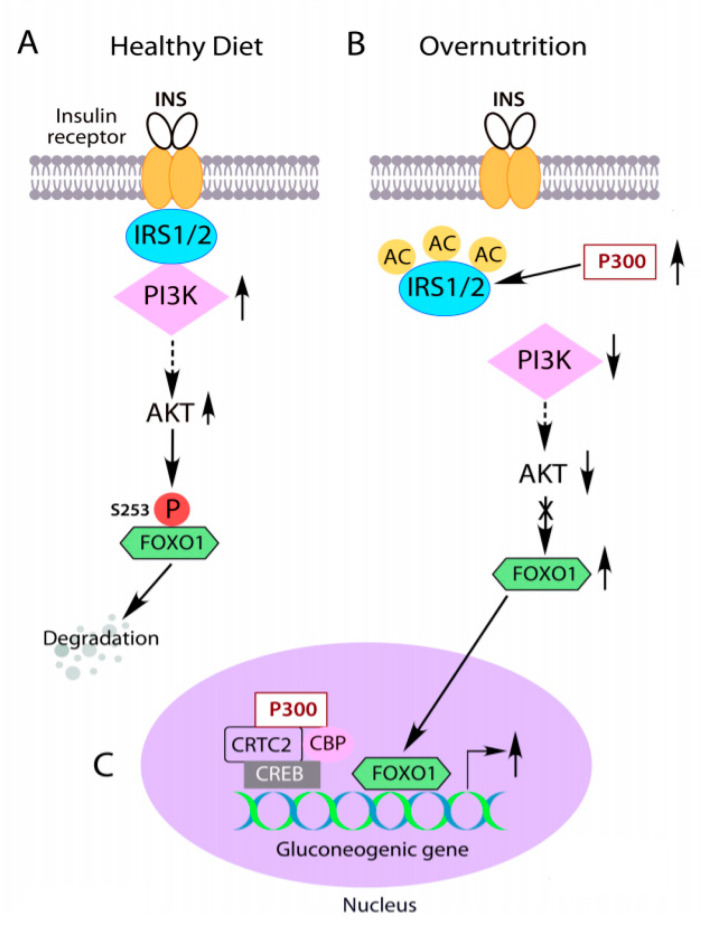
IRS acetylation by abnormal cytoplasm-appearing P300 causes insulin resistance. (**A**) Insulin-mediated activation of PI3K-AKT signaling leads to FOXO1 phosphorylation, nuclear exclusion and degradation, subsequently inhibition of gluconeogenic gene expression in the liver. (**B**, **C**) Overnutrition induced abnormal cytoplasm-appearing P300 acetylates IRS1 and IRS2 to disrupt their association with insulin receptors and insulin signaling. FOXO1 cannot be phosphorylated by AKT (**B**), leading to its nuclear accumulation and stimulation of overexpression of the gluconeogenic gene in the liver (**C**). The solid arrows indicate the direct effects, the dashed arrows indicate indirect effects, and the crossed line indicates the blockade of the pathway.

Insulin binds to the extracellular α-subunit of the insulin receptor (IR), causing autophosphorylation of the membrane-bound β-subunit of IR by its intrinsic tyrosine kinase activity [69,70]. Phosphorylation of the IR at Y972 by insulin increases both affinity of insulin receptor substrate (IRS) proteins binding to the IR and tyrosine phosphorylation of IRS [71]. IRS binds to the plasma membrane and IR through the pleckstrin-homology domains (PH domain) and phosphotyrosine-binding domains (PTB domain), respectively [72,73,74,75,76]. The phosphorylation of tyrosine residues in the IRS by the IR, recruits p85 subunit in PI3K to IRS, and activation of the PI3K-AKT signaling cascade, resulting in the suppression of hepatic glucose production (Figure 2A) [41,55,77,78]. Mice with liver-specific double IRS1 and IRS2 knockout exhibit severe hyperglycemia [79,80], suggesting that liver IRS1 and IRS2 are the critical mediators of insulin’s regulation of glucose metabolism. Several lines of evidence show that the impairment of proximal insulin signaling causes insulin resistance in obesity and T2D [80,81].

Insulin resistance, the hallmark of T2D, leads to increased liver glucose production and decreased glucose utilization in extrahepatic tissues, such as muscle and adipose tissues, resulting in fasting hyperglycemia in patients with T2D [82,83]. While postprandial glucose cannot be effectively converted and stored as glycogen in the liver, this accentuates the overproduction of glucose in the liver. Moreover, liver insulin resistance can cause systemic insulin resistance [84]. Overnutrition with excess caloric load results in glucose uptake in the muscle that exceeds its handling capacity, and excess glucose returns and spills into de novo lipogenesis and causes steatosis in the liver. With the failure of insulin action in adipose tissues, more free fatty acids will be released and shuttled to the liver. This would aggravate liver steatosis and insulin resistance in the liver [85].

### 5.1. LPS-Induced P300 Impairs Insulin Signaling

Increased LPS leakage from the gut into the circulation and endoplasmic reticulum (ER) stress play important roles in the development of insulin resistance in obesity and T2D [35,85,86,87]. Since HFD feeding elevated LPS levels, we tested LPS effects on insulin signaling and found that LPS treatment significantly decreased AKT and GSK phosphorylation by insulin [59]. LPS augmented P300 protein levels in the cytoplasm and ER stress [59]. To assess whether P300 could affect insulin signaling, we used adenoviral shRNA to deplete P300 in Hepa1-6 cells and found that depletion of P300, but not its closely related protein CBP, increased insulin-mediated AKT and GSK phosphorylation. Furthermore, in a hyperinsulinemic-euglycemic clamp experiment, shRNA-mediated depletion of 85% of liver P300 (remaining P300 protein levels are similar to that of liver P300 in mice fed a regular diet) in mice fed an HFD improved liver insulin sensitivity, suggesting that HFD-induced P300 is an important pathological factor leading to the development of liver insulin resistance in obesity.

### 5.2. Acetylation of IRS1 and IRS2 by Abnormal Cytoplasm-Appearing P300 Impairs Insulin Signaling

P300 has an intrinsic acetyltransferase activity [44,45], and its acetyltransferase activity may affect insulin signaling. To test this hypothesis, curcumin, a specific inhibitor of both P300 and CBP acetyltransferase activity, was employed to treat Hepa1-6 cells. Curcumin treatment significantly increased AKT and GSK3 phosphorylation. Pre-treatment with the P300 acetyltransferase-specific inhibitor C646 led to a significant increase in AKT and GSK3 phosphorylation levels, both in the absence and presence of insulin, compared to treatment with the control inactive compound C37 [59]. In HFD-fed mice, treatment with C646 for 2 weeks significantly improved insulin sensitivity without the significant change of body weight [59]. Intriguingly, C646 treatment without insulin not only increased AKT and GSK3 phosphorylation and PI3K activity but also caused mobility shifts of IRS, which is often associated with tyrosine phosphorylation of these proteins. Indeed, C646 treatment significantly increased tyrosine phosphorylation of IRS1 and IRS2 but had a minimal effect on IRβ phosphorylation at Y972 [59]. In lieu of these findings, we treated *ob/ob* mice with inhibitor C646 because *ob/ob* mice have significantly increased levels of liver P300 protein and exhibit significantly increased acetylation of liver IRS1 and IRS2. Treatment with C646 significantly decreased IRS acetylation levels and significantly increased tyrosine phosphorylation of IRS1 and IRS2 proteins in the liver of *ob/ob* mice [59]. These data substantiate the role of P300 acetyltransferase activity in the impairment of insulin signaling in obesity and indicate that abnormal cytoplasm-appearing P300 impairs insulin signaling by acetylating IRS1 and IRS2 (Figure 2B).

Further studies revealed that seven lysine residues in IRS1 and 15 in IRS2 could be acetylated. In particular, the acetylation of lysine residues at 1017/1080/1131 in IRS1 and lysine residues at 1173/1264 in IRS2 have the strongest impacts on insulin signaling. The expression of mutated IRS1-K1017/1080/1131R plus IRS2-K1173/1264R mutated proteins significantly improved insulin sensitivity in HFD-fed mice [59]. In accord with inhibition of P300 acetyltransferase activity improves insulin signaling, activation of the deacetylase Sirtuin 1 restores insulin sensitivity in tissues with insulin resistance through deacetylation [88,89,90,91,92,93]. In contrast, inhibition of deacetylase activity increased IRS2 acetylation and reduced IRS2 tyrosine phosphorylation [94]. These data suggest that P300 may be the acetyltransferase that counter-regulates Sirtuin 1′s deacetylase activity. However, IRS1 acetylation has a permissive impact on its tyrosine phosphorylation, and IRS1 and IRS2 can be acetylated by other acetyltransferases at different sites [95,96]. These acetylation sites may have different effects on insulin signaling.

### 5.3. Low-Grade Inflammation Can Disrupt Insulin Signaling through IRS Serine/Threonine Phosphorylation

Association of LPS to CD14 and TLR4 induces MyD88-dependent signaling from the cell surface and activates the NFkB signaling pathway [97,98]. The activation of NFkB signaling would augment IKKβ-mediated phosphorylation of multiple serine/threonine sites in IRS [99,100]. Furthermore, IKKβ activation could increase serine phosphorylation of IRS through activation of the mTORC1/P70S6K pathway [99,100]. The phosphorylation of serine and threonine residues in IRS has a negative impact on insulin signaling and contributes to pathological insulin resistance [80,83]. Furthermore, activation of ER stress by LPS results in the formation of the IRE1α-TRAF2 complex, which leads to the phosphorylation and activation of c-Jun N-terminal kinase (JNK). Activated JNK, in turn, mediates broad IRS serine/threonine phosphorylation to cause insulin resistance [101,102].

### 5.4. Changes in Gut Microbiota by Overnutrition Silence Enteroendocrine Cells

Hormones secreted from enteroendocrine cells play critical roles in the regulation of nutrient metabolism and insulin sensitivity. These hormones, such as GLP-1 and GIP, have been used as therapeutics for obesity and T2D. However, HFD feeding reduced intestinal glucose sensing and glucose-induced GLP-1 secretion in mice [103]. The cultured small intestine from HFD-fed mice displayed a reduced glucose-stimulated secretory response, including GLP-1 release from L cells [104]. A later study revealed that HFD feeding could alter the morphology of enteroendocrine cells and convert them into a nutrient insensitive state by HFD-enriched *Acinetobacter* bacteria [105]. SCFAs can trigger the release of GLP-1 from L cells [106]; thus, the reduction of SCFAs by HFD feeding would be another explanation of decreased secretion of GLP-1.

## 6. Perspective

Disruption of the microbiota community living in the intestine will increase the risk and severity of the host’s medical conditions. Maintaining a healthy gut microbiota is important for reducing the risk of developing disorders. In healthy adults, the gut’s microbial community is relatively stable and composed of highly adapted microbial species and has been shaped more by environmental and lifestyle factors than host genetics [107,108]. A Western diet with high fat content can change the gut’s microbial profile and reduce its diversity [15,16]. Since diet-induced changes in gut microbiota are reversible [20], dietary intervention or modulation of the gut microbiota by probiotic microorganisms can restore gut microbiota structure and functions [109,110,111]. Precision microbiota study can pinpoint personalized diet to restore a healthy and diverse gut microbiota. The gut microbiota dysbiosis in obesity and T2D is associated with increased gut permeability (leaky gut). Strengthening tight junctions and mucin formation can reduce LPS uptake from the intestine. Food-derived interventions, including the supplementation of SCFAs, can be developed to regulate microbiota and strengthen tight junctions to treat or prevent disease in T2D and obesity (Figure 1A). Of note, metformin, a first-line antidiabetic agent, can reach extremely high concentrations (1.3 mM) in the intestine after oral administration [112,113]. These high concentrations can alter gut microbiota and maintain the integrity of the intestinal barrier by strengthening the tight junctions via AMPK activation [114,115,116,117].

It is well known that LPS can trigger ER stress, and alleviation of ER stress negates the activation of the IRE1-XBP1 pathway and P300 induction by LPS [59,61]. P300 could not be induced in the absence of CD14 [59], suggesting that LPS-mediated P300 is through the CD4/TLR4 signaling pathway (Figure 1B). However, the downstream mediators linking CD4/TLR4 to ER stress needs to be delineated in future studies. Activation of ER stress by LPS can impair insulin signaling through IRS acetylation by induced P300 and serine phosphorylation by JNK. It is possible that IRS acetylation may exacerbate IRS serine phosphorylation or vice versa. Insulin resistance will decrease FOXO1 phosphorylation by insulin through PI3K-AKT, which would retain FOXO1 in the nucleus and prevent cytoplasmic ubiquitinoylation and degradation, resulting in increased expression of *G6pc* and *Pck1* (Figure 2B,C) [73,118,119,120]. CRTC2 may undergo similar retention in the nucleus as S171 cannot be phosphorylated by AKT under the condition of insulin resistance and augments gluconeogenic gene expression in the liver [41]. Moreover, LPS-initiated low-grade inflammation and ER stress can cause insulin resistance in muscle and adipose tissues and a decrease in glucose utilization in these tissues [83].

Curcumin, an inhibitor for both P300 and CBP acetyltransferase activity, has been used to alleviate hyperglycemia in diabetic patients and animal models [121,122,123]. A-485, a more potent and specific inhibitor of both P300 and CBP acetyltransferase activity, alleviated fasting blood glucose levels in HFD-fed mice [66]. C646, a P300 acetyltransferase-specific inhibitor, significantly improved hyperglycemia and improved insulin sensitivity in obese mice [59]. These data suggest that P300 acetyltransferase activity is a therapeutic target for the alleviation of hyperglycemia and insulin resistance in T2D and obesity. However, P300 is an important co-activator involved in chromatin remodeling and participates in many critical developmental processes. Undoubtedly, developing a drug specifically targeting P300 functions in the regulation gluconeogenic gene expression and insulin signaling is of importance in the treatment of T2D and obesity.
